# Large retroperitoneal mass: A case of an undifferentiated pleomorphic sarcoma

**DOI:** 10.1016/j.radcr.2024.09.141

**Published:** 2025-01-15

**Authors:** Millennie J. Chen, Shafieh Makehei, Isaac Chen, Simona De Michele, Shiv Bhanu, Justin Wei

**Affiliations:** Riverside Community Hospital, 4445 Magnolia Ave, Riverside, CA 92501, USA

**Keywords:** Undifferentiated pleomorphic sarcoma, Soft tissue sarcoma, Retroperitoneal mass, Resection, Histopathology

## Abstract

Soft tissue sarcomas (STS) are a group of rare malignant tumors arising from mesenchymal stem cells. There are more than 60 different types of neoplasms that fall under the umbrella of STS, including tumors that originate from cartilage, adipose tissue, skeletal muscle, or connective tissue, among many other tissue types. One particular type of high-grade aggressive STS is the undifferentiated pleomorphic sarcoma (UPS), formerly known as the malignant fibrous histiocytoma (MFH). There has historically been much debate about the classification and differentiation of UPS from other types of sarcomas, which has only recently been characterized by novel methods of immunohistochemistry markers and sophisticated cytogenetics. This has left much of the literature regarding UPS largely irrelevant in context of our current classification standards.

A recent retrospective analysis of 100 UPS cases revealed an incidence of only 9% for UPS originating in the retroperitoneum. We present a case of UPS originating in the retroperitoneum. In this case, a patient suffering from a large right-sided intra-abdominal mass underwent complete surgical resection. We also demonstrate some of the complexities involved in the diagnosis and treatment of a rare retroperitoneal form of UPS.

## Introduction

Soft tissue sarcomas (STS) are a group of rare malignant tumors arising from mesenchymal stem cells [1]. One particular type of high-grade aggressive STS is the undifferentiated pleomorphic sarcoma (UPS), formerly known as the malignant fibrous histiocytoma (MFH) [2]. These masses can originate from various locations throughout the body, including the extremities, trunk, and retroperitoneum [[1], [3]]. The pathologic mechanisms that drive the formation of UPS is not well understood, and there are no specific defining features other than lack of an identifiable line of differentiation [4].

For UPS, it is preferable to obtain sampling via the core needle technique over the open incisional biopsy technique due to the increased risk of hemorrhage and the additional incision that is made [2]. Fine needle aspiration is also not ideal due to insufficient sampling. For patients whom none of the above techniques are safe and viable, complete resection may be performed and the entire mass may be utilized for histopathological investigation.

There has historically been much debate about the classification and differentiation of UPS from other types of sarcomas and cancers, and current literature regarding UPS is limited, as many prior papers citing this tumor are either using an outdated classification system for sarcomatous tumors or have classified UPS as MFH [5]. Given the rarity of primary retroperitoneal UPS, with few case reports in the current literature, we highlight here some of the imaging features in 1 such case.

## Case presentation

An 88-year-old female presented to the emergency room with recent iron deficiency anemia, fatigue, occasional vague abdominal pain, and 10 lbs of unintentional weight loss over the past few months. Her past medical history was otherwise unremarkable. Her family history was significant for prostate cancer in her brother and unspecified cancer in her sister.

**Fig. 1:**
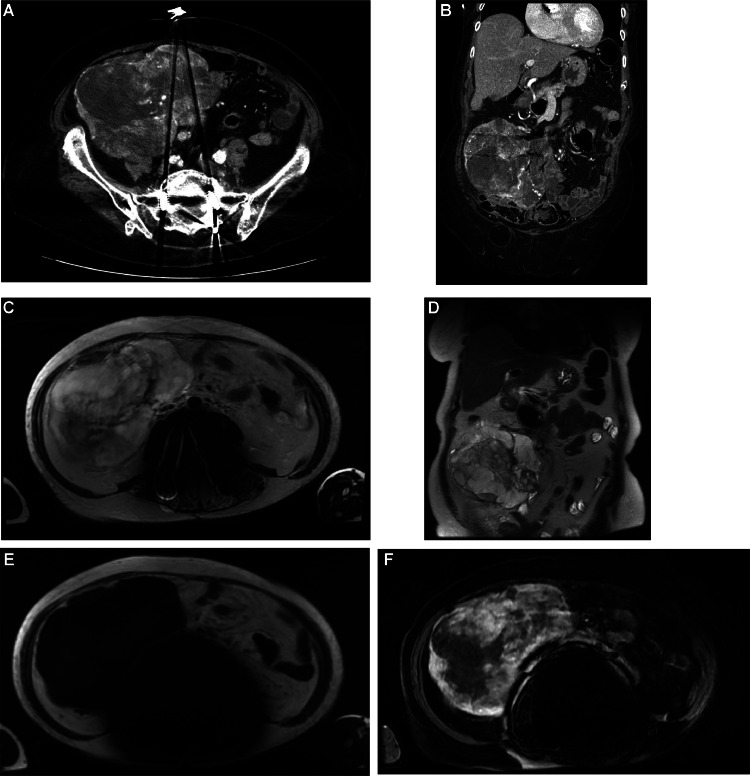
(A and B) Axial & coronal CT abdomen/pelvis demonstrating a large heterogeneous partially vascular and necrotic mass measuring 11.0 × 15.7 × 15.5 cm within the right lower quadrant of the abdomen/mesentery. The mass extends from the anterior pararenal space and compresses the ascending colon anteriorly. (C and D) Axial & coronal T2 weighted MRI abdomen showing diffuse heterogeneous hyperintense signal. (E and F) Axial T1 & T1 weighted fat saturated postcontrast MR images demonstrating prominent peripheral enhancement of the mass.

CT imaging obtained in the hospital revealed a large heterogeneous partially necrotic right lower quadrant (RLQ) abdominal/mesenteric mass containing multiple vessels. This mass extended adjacent to the inferomedial aspect of the right hepatic lobe and adjacent to the junction of the second and third segments of the duodenum, with complete effacement of the majority of the ascending colon and with anterior displacement of the proximal ascending colon and portions of the cecum (Fig. 1A and 1B). As such, involvement of the distal small bowel or ascending colon was considered a possibility. Due to the apparent mechanism of ascending colon displacement, a retroperitoneal origin was considered more likely, namely in the anterior pararenal space. Further, there was no apparent extension to the right adnexa and uterus, so a genitourinary origin was deemed unlikely. Differentials at the time included various large mesenteric masses, a large gastrointestinal stromal tumor (GIST), or an enlarged mesenteric lymph node.

Further evaluation with MRI was obtained, which similarly showed the large RLQ abdominal mass abutting the ascending colon/cecum, with heterogeneous T2 hyperintense, T1 isointense/hypointense signal, and a predominantly peripheral enhancement pattern (Fig. 1C, 1D, 1E, and 1F). Significant anterior displacement of the ascending colon and leftward displacement of small bowel was demonstrated. No significant signal dropout was demonstrated on in versus out of phase images, indicating a lack of significant fatty content. Susceptibility artifact prevented adequate evaluation with DWI and ADC sequences. Due to the location of the mass, it was unclear whether the origin of the tumor was colonic or mesenchymal in origin. Accordingly, differentials here included various malignancies, namely colon cancer or a mesenteric tumor like sarcoma, with GIST considered less likely.

**Fig. 2:**
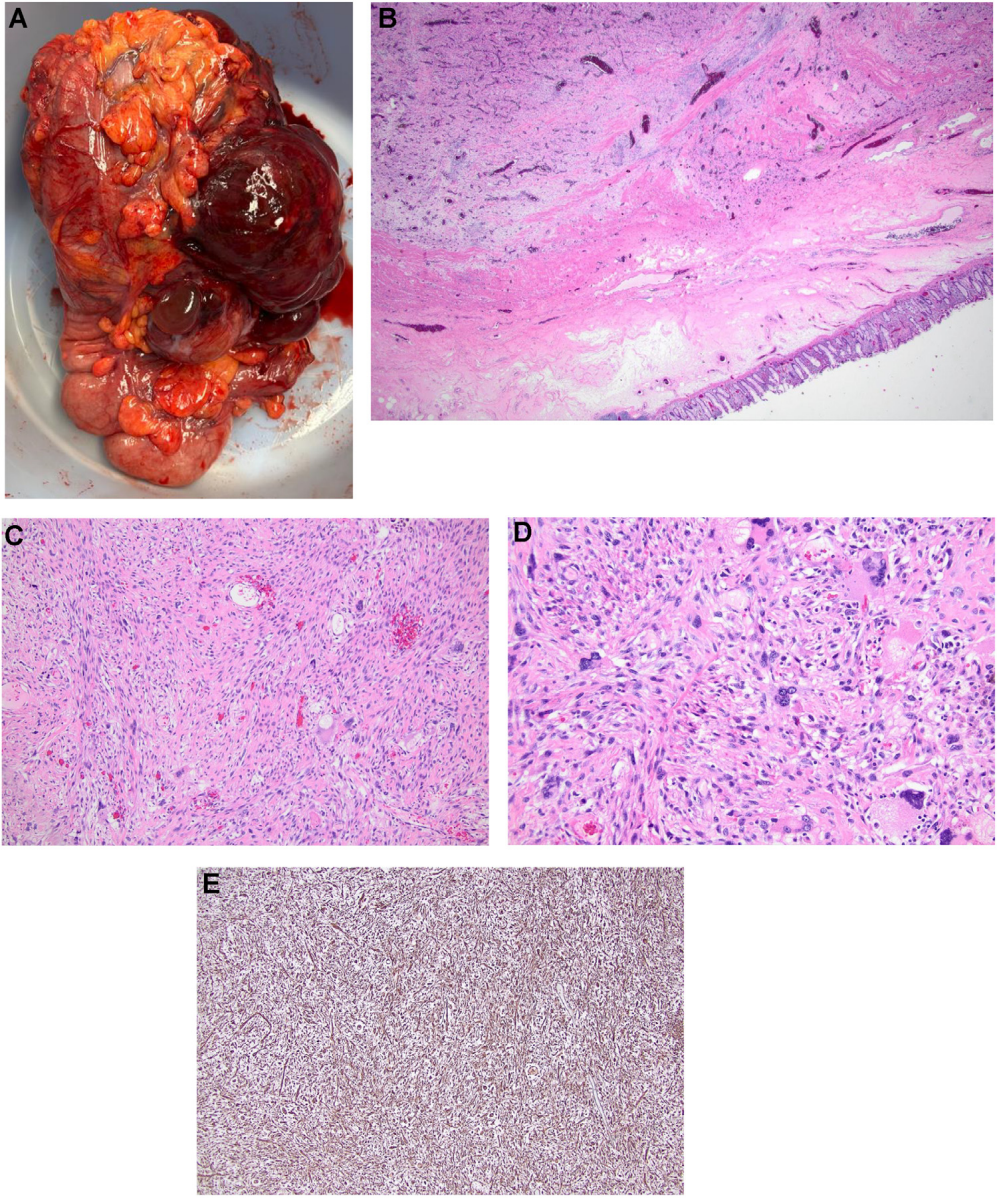
(A) Gross specimen of undifferentiated pleomorphic sarcoma. (B) Undifferentiated pleomorphic sarcoma (UPS) involving colonic wall (H&E, x2). (C) At medium power, spindle nuclei are scattered among larger cells growing in a vague storiform pattern (H&E, x10). (D) At high power, many atypical, bizarre cells with increased nuclear to cytoplasmic ratio and hyperchromasia are noted (H&E, x20). (E) The tumor shows strong and diffuse immunoreactivity for Vimentin, a nonspecific marker of mesenchymal origin, typically positive in UPS (Vimentin, x4).

In this case, percutaneous biopsy was forgone in favor of exploratory laparotomy with mass excision and right hemicolectomy, due to concern for an elevated risk of bleeding and tumor seeding. The patient underwent complete resection of the mass, as well as segments of the right colon and distal small bowel en bloc. Gross evaluation revealed a large multilobulated mass measuring 15.5 × 12.4 × 9.0 cm, focally involving the right colon with transmural extension up to the mucosa with ulceration (Fig. 2A and 2B). The cut section showed a heterogeneous tan-gray tumor with fleshy areas, abundant hemorrhage and necrosis. Microscopically, the tumor showed a patternless arrangement of hyperchromatic spindle to epithelioid neoplastic cells with abundant highly pleomorphic and bizzare/multinucleated cells with amphophilic cytoplasm and high mitotic rate (Fig. 2C and 2D). Frequent areas of tumor necrosis were identified. The immunohistochemistry workup was negative for any distinct lineage marker, while only positive for CD34 and vimentin (Fig. 2E). The margins of resection were negative for sarcoma, with the closest margin being 3.2 cm away from the tumor. MDM2 fluorescence in situ hybridization analysis did not show MDM2 amplification. Next-generation sequencing analysis was also negative.

The patient was retained in the hospital for 4 days after the operation, after which she reported general resolution of her symptoms and pain.

## Discussion

Of UPS lesions detected in the abdominal cavity, most originate from the kidney and most occur in male patients [4]. Retroperitoneal UPS tumors in the anterior pararenal space & abutting the colon are more rare, and, to our knowledge, have not yet been described in detail. The case presented in this study holds particular significance as it demonstrates a rare and challenging case of a UPS originating in the retroperitoneum in a female patient.

The differential diagnosis for large intra-abdominal masses includes various benign and malignant conditions. In this case, the mass exhibited significant necrosis and neovascularity, which are characteristic features of malignancy. During early workup of this patient's tumor, the location and radiologic features of the mass suggested a possible gastrointestinal origin involving the ascending colon or cecum. However, the tumor could have not been characterized without histopathological evaluation, which revealed a tumor composed of pleomorphic spindle shaped and epithelioid cells, including highly atypical forms, with no osteoid or cartilage production and without identifiable line of differentiation. Of note, positivity for CD34 has been suggested as a positive prognostic factor [6].

The management of UPS remains a dynamic field. Future larger-scale studies that explore the safety of different diagnostic and treatment modalities are needed to confirm the optimal management of retroperitoneal UPS on a larger patient scale. Furthermore, this case report does not include long term follow-up of this patient, which means the long term prognosis of a patient with a retroperitoneal UPS cannot be exemplified in this case like it has been for other types of sarcomas. Continued efforts for ongoing research and clinical experience are crucial to enhancing prognostic value and developing more targeted therapies for this aggressive malignancy.

## Conclusion

Our case report demonstrates some of the complexities involved in the diagnosis of a rare retroperitoneal form of UPS, which requires correlation with clinical, radiological and pathologic findings. Comprehensive imaging, as well as further diagnostic steps, such as biopsy or surgical resection, is essential for definitive diagnosis and treatment planning of rare cancers like UPS. Future cases describing masses of similar origin in the retroperitoneum would be important to gain a more comprehensive understanding of the imaging characteristics of these high-grade and aggressive tumors.

## Patient consent

Written, informed consent for publication of this patient's case was obtained from the patient.

## References

[1] Popovich JR, Kashyap S, Gasalberti DP, Cassaro S Sarcoma. StatPearls. Treasure Island, FL:StatPearls Publishing; 2024. Accessed August 10, 2024 http://www.ncbi.nlm.nih.gov/books/NBK519533/.

[2] Robles-Tenorio A, Solis-Ledesma G Undifferentiated pleomorphic sarcoma. StatPearls. Treasure Island, FL:StatPearls Publishing; 2024. Accessed August 10, 2024 http://www.ncbi.nlm.nih.gov/books/NBK570612/. 34033374

[3] Chen S, Huang W, Luo P (2019). Undifferentiated pleomorphic sarcoma: long-term follow-up from a large institution. Cancer Manag Res.

[4] Yu K, Wang L, Bu F, Zhang J, Hai R, Lu J (2023). Retroperitoneal undifferentiated pleomorphic sarcoma with total nephrectomy: a case report and literature review. Front Surg.

[5] Karki B, Xu YK, Wu YK, Zhang WW (2012). Primary malignant fibrous histiocytoma of the abdominal cavity: CT findings and pathological correlation. World J Radiol.

[6] Sugiura Y, Machinami R, Matsumoto S, Kanda H, Ae K, Takazawa Y (2021). Prognostic value of CD34 expression status in patients with myxofibrosarcomas and undifferentiated pleomorphic sarcomas. Sci Rep.

